# Effect of Ti on Microstructure and Properties of Tungsten Heavy Alloy Joint Brazed by CuAgTi Filler Metal

**DOI:** 10.3390/ma12071057

**Published:** 2019-03-30

**Authors:** Yuzhen Lu, Xiaoming Qiu, Ye Ruan, Cui Luo, Fei Xing

**Affiliations:** 1 Key Laboratory of Automobile Materials of Ministry of Education, School of Materials Science and Engineering, Jilin University, Changchun 130022, China; luyz16@mails.jlu.edu.cn (Y.L.); qiuxm16@hotmail.com (X.Q.); ruanye@jlu.edu.cn (Y.R.); luocui16@mails.jlu.edu.cn (C.L.); 2 Key of Laboratory of Automotive Simulation and Control, Jilin University, Changchun 130022, China

**Keywords:** tungsten heavy alloy, brazed, active element, Ti

## Abstract

In this paper, scanning electron microscopy (SEM), energy dispersive spectroscopy (EDS) and X-ray diffractometer (XRD) were used to comprehensively analyze the microstructure and brazing performance of a CuAgTi filler metal with braze tungsten heavy alloys. The association of microstructure, wettability and shear strength of brazing joints was also investigated. With the addition of Ti, the Ti_3_Cu_4_ phase appeared in the microstructure of filler metal. Ti is active element that promotes the reaction of filler with tungsten. Therefore, the Ti element is enriched around tungsten and forms a Ti_2_Cu layer at the interface, leaving a Cu-rich/Ti-poor area on the side. Remaining Ti and Cu elements form the acicular Ti_3_Cu_4_ structure at the center of the brazing zone. The wettability of filler metal is improved, and the spreading area is increased from 120.3 mm^2^ to 320.9 mm^2^ with the addition of 10 wt.% Ti. The shear strength of joint reaches the highest level at a Ti content of 2.5 wt.%, the highest shear strength is 245.6 MPa at room temperature and 142.2 MPa at 400 °C.

## 1. Introduction

Tungsten heavy alloys are composite materials while W is used as the hard phase and (Ni, Cu), (Ni, Fe) are used as the binder phases [1,2]. Tungsten heavy alloy is one of the most attractive metals for the nuclear industry, turbine and rocket power systems and machinery manufacturing applications due to their excellent properties such as high strength, good thermal conductivity, low thermal expansion coefficients and high corrosion resistance [3,4]. However, Tungsten heavy alloy is produced by a powder metallurgy process which is difficult to be machined, resulting in the urgently need for welding to form large or complex structures. The poor weldability of tungsten heavy alloy restricts the development and application in tungsten heavy alloy components.

Tungsten and its alloys tend to cause incomplete fusion during fusion welding because of their high melting point and thermal conductivity. The joining of tungsten alloys has been achieved by several procedures: brazing [5,6,7], diffusion bonding [8,9,10] and laser brazing [11]. Among them, high temperature brazing seems to be the most suitable method because of its limited influence on properties of base materials. In order to braze tungsten alloy, various components of filler metals have been investigated, such as Fe-based filler metals [12,13], Ni-based filler metals [14,15] and Cu-based filler metals [16,17]. However, when the brazing temperature is above the recrystallization temperature of W (1100–1370 °C), performance changes of base metals such as grain growth, harden, and embrittlement could occur because of the high melting point of filler metals [18]. Cu-Ag alloys have been widely applied as brazing filler metals due to their superiority of strength, plasticity and weldability. Ti is active element which is investigated as reactive filler metals for brazing of ceramics [19,20,21] and cemented carbide [22,23] which are difficult to weld. In addition, Ti has a higher solubility in W. Researchers have tried to extend the application of Ti to braze of tungsten alloys. Chiachen Lin et al. studied Pd and Ti foils with different thicknesses, for brazing of tungsten and molybdenum in vacuum at temperatures between 1580 and 1740 °C for 10 min, the result shows that brazed joint by Ti foils is full of voids/cracks. The reason for this is that the dissolution of W into the filler metal causes an increase in the solidus and liquidus temperatures of filler metal, so there is insufficient liquid to fill up the joint [24]. J. de Prado et al. studied Fe-Ti filler metal for W-W brazing joint which brazed in a high vacuum furnace at 1350 °C for 10 min, the shear strength of the joints is 94 ± 23 MPa, and failure during testing is always located at the tungsten-braze interface, because no further reactions or interactions were observed at the W-braze interface [25].

Considering the problem that the brazing temperature of the tungsten-based powder alloy using Fe-based or Ni-based brazing filler metal is higher than the recrystallization temperature of tungsten, this paper used a Cu-30Ag base filler metal and added a Ti element to promote its interaction with base metal. The research of Shang J has proved that the Cu-69.5Ag-3.5Ti filler metal had good wettability on the pure W plate, which has the same main component as the tungsten-based powder alloy [26]. The addition of Ti will produce intermetallic compounds in the joint, and excessive Ti will reduce the mechanical properties of the joint. Therefore, this paper further studied the influence of Ti on the microstructure and mechanical properties of the joints, and found the most suitable Ti content to provide a basis for optimizing the filler composition.

## 2. Materials and Methods 

**Figure 1:**
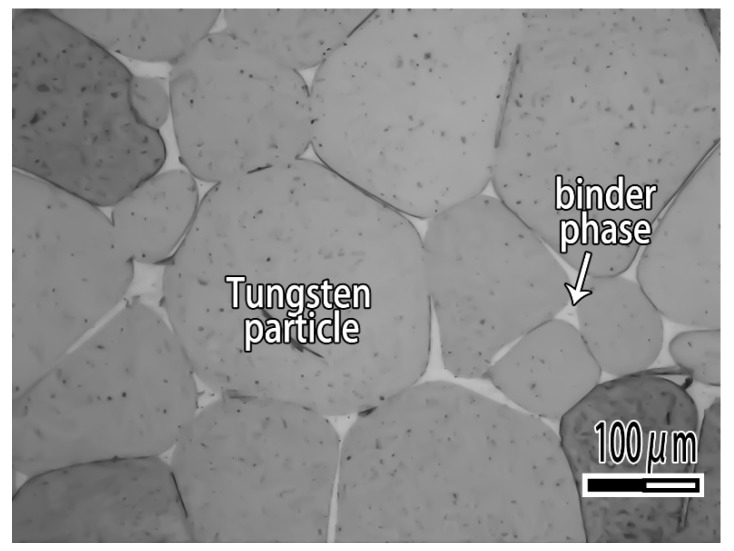
Microstructure of tungsten heavy alloy.

A tungsten heavy alloy plate used in the study is made by 97 wt.% W, 1.7 wt.% Ni and 1.3 wt.% Fe. As can be seen from Figure 1, tungsten heavy alloy is composite material which consisted of tungsten particle and binder phase. The plates are treated with the surface preparation consisting of mechanical polishing with 1000 grit SiC abrasive followed by an ultrasonic cleaning in acetone, rinsed successively in ethanol and then dried with warm air. CuAgTi filler metals is prepared by mixing of Cu, Ag and Ti metal powders according to the ratio of Table 1, then smelting in TL1400 high-vacuum furnace (TL1400, Nanjing Boyuntong Instrument Inc., Nanjing, China) at a maximum pressure of 7.6 × 10^−3^ Pa, at 1050 °C holding for 30 min. 

**Figure 2:**
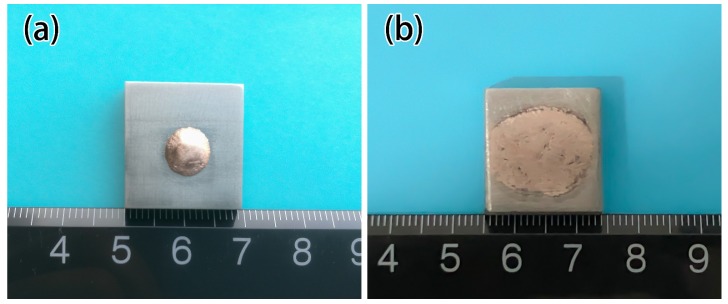
Wettability sample of CuAgTi filler metal. (**a**) Ti 1 wt.%; (**b**) Ti 7.5 wt.%.

**Figure 3:**
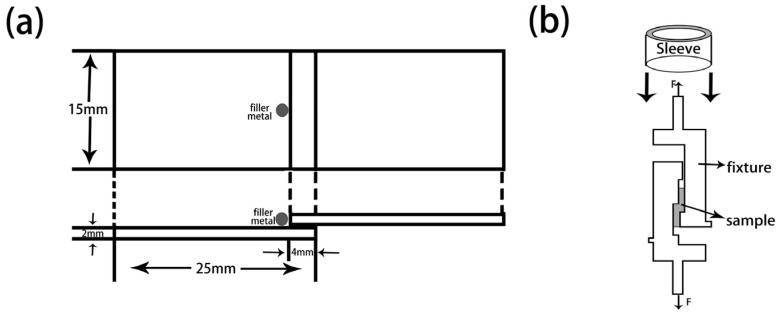
Schematic diagram. (**a**) Brazing joints; (**b**) Shear strength tests.

The wetting experiments are carried out on the tungsten heavy alloy plates (20 mm (in Width) × 20 mm (in Length) × 2 mm (in Thickness)) with a similar ball filler of 0.30 g placed on it. The wetting experiment samples are produced in TL400 high-vacuum brazing furnace at 1100 °C holding for 10 min with a pressure of 5 × 10^−3^ Pa. Take the sample out and measure the spreading area after solidification and cooling to room temperature. The prepared wetting samples are shown in Figure 2. The shear strength experiments samples are processed the two matrix plates overlap with the dimension of 30 mm (in Length) × 15 mm (in Width) × 2 mm (in Thickness), as shown in Figure 3a. Dead load of 20 g is applied on top of the overlap joint assemblies to provide a slight bonding load. Then we placed them in a vacuum furnace and heated them at the same temperature and pressure as for wetting experiments. The shear strength tests are performed at ambient and high temperature on an electronic universal experimental machine (INSTRON-5869, INSTRON Inc., Norwood, MA, USA) at a constant speed of 1 mm/min with the aid of a fixture. The schematic diagram of shear strength tests is shown in Figure 3b. The microstructures, element distribution and fracture morphology analysis of the brazed samples were performed using Micro X-ray diffractometer (XRD, D8 Discover with GADDS, BRUKER, Karlsruhe, Germany) and scanning electron microscopy (SEM, S-3400N, HITACHI, Kyoto, Japan) equipped with an energy dispersive spectroscopy (EDS, Genesis XM, EDAX Inc., Mahwah, NJ, USA). 

## 3. Results and Discussions

### 3.1. Filler Metal

**Figure 4:**
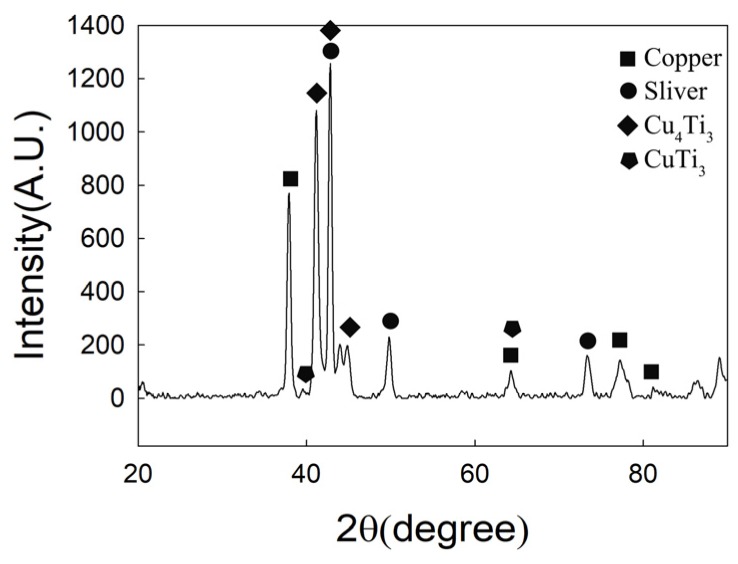
XRD Pattern of CuAgTi filler metals.

**Figure 5:**
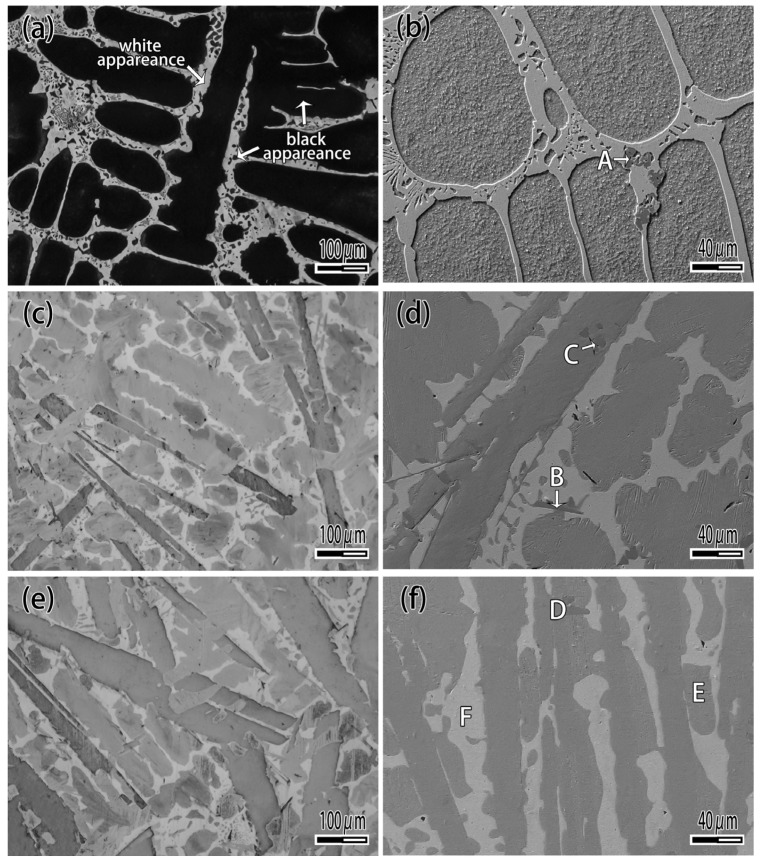
Microstructure of CuAgTi filler metal. (**a**) 1 wt.% Ti; (**b**) Magnified of 1 wt.% Ti; (**c**) 7.5 wt.% Ti; (**d**) Magnified of 7.5 wt.% Ti; (**e**) 10 wt.% Ti; (**f**) Magnified of 10 wt.% Ti.

Figure 4 is the XRD pattern of CuAgTi filler metal, it shows that the filler metals consist of α-Cu, β-Ag and a small amount of intermetallic phases Ti_3_Cu_4_. Figure 5 exhibits the microstructure of CuAgTi filler metals with different Ti content. It can be seen that the microstructure is similar to Cu-Ag binary alloy (Figure 5a), and consisted of black α-Cu and white β-Ag while the Ti content is less than 2.5 wt.%. When Ti content exceeds 7.5 wt.% (Figure 5c,e), a rectangular structure appeared, which was augment with the increasing of the Ti content. XRD and EDS (Table 2) reveal that the Ti element aggregates in the form of Ti solidus solution at the junction of copper and silver, as shown by point A in Figure 5b. With the increasing of the Ti content, the rectangular structure that appeared is the Ti_3_Cu_4_ intermediate phase, which is marked B in Figure 5d, and the Ti element mainly exists in this form. When the Ti content is 10 wt.%, the filler metal consisted of white β-Ag matrix (point F), oval α-Cu (point E) and rectangular Ti_3_Cu_4_ phase (point D) in Figure 5f.

**Figure 6:**
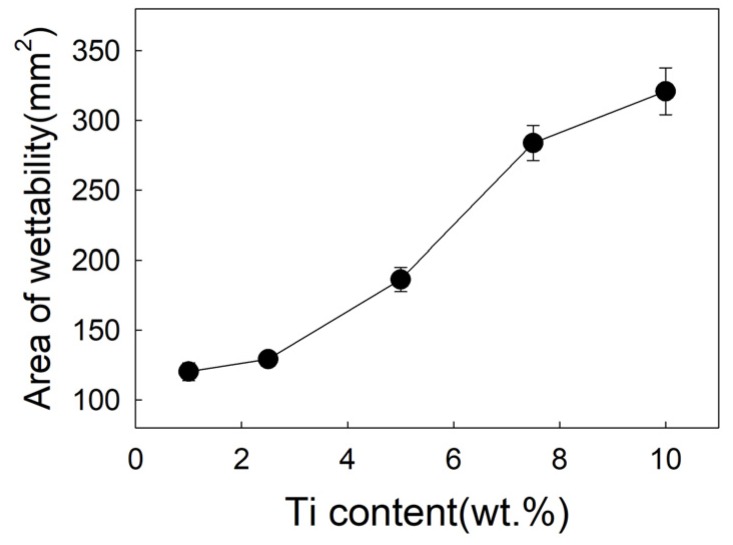
Influence of Ti content on spreading area.

Figure 6 exhibits the effect of Ti on the spreading area of filler metal. In test range, the spreading area of the filler is 120.31 mm^2^ with 1 wt.% Ti added, as shown in Figure 2a. With 5 wt.%Ti added, the spreading area rapidly increased to 186.3 mm^2^, and reached 283.8 mm^2^ with 7.5 wt.% Ti added, as shown in Figure 2b. When Ti content is 10wt.%, the spreading area is 40mm^2^ which is more than 7.5 wt.%. As shown in Figure 2, when the Ti content is 7.5 wt.%, the spreading area is much larger than the Ti content of 1wt.%, the wettability is significantly improved. It can be seen that while the Ti content is 7.5 wt.%, the microstructure of filler metal changes, forming a rectangular Ti_3_Cu_4_ phase, the spreading area obviously also increased to 283.8 mm^2^.

### 3.2. Microstructure of brazing joint

**Figure 7:**
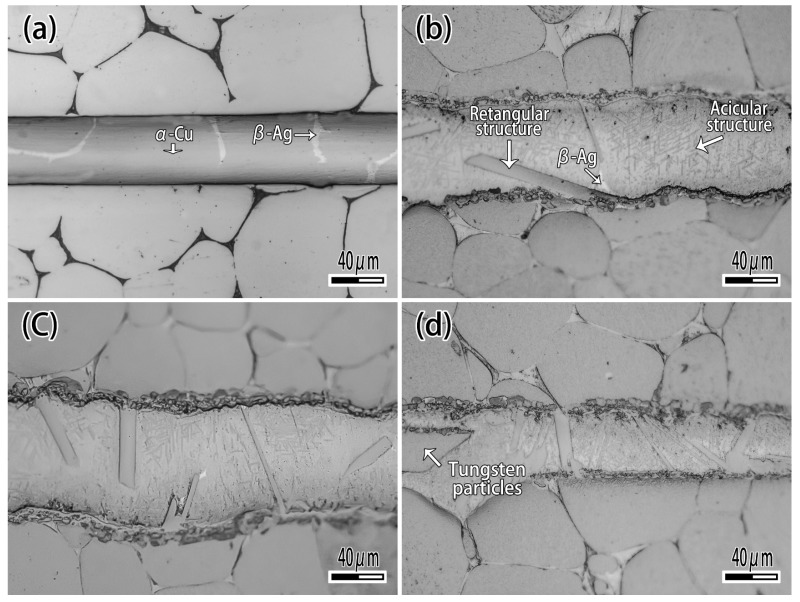
The effect of Ti on microstructure of joint. (**a**) Ti 1 wt.%; (**b**) Ti 2.5wt.%; (**c**) Ti 5 wt.%; (**d**) Ti 10 wt.%.

**Figure 8:**
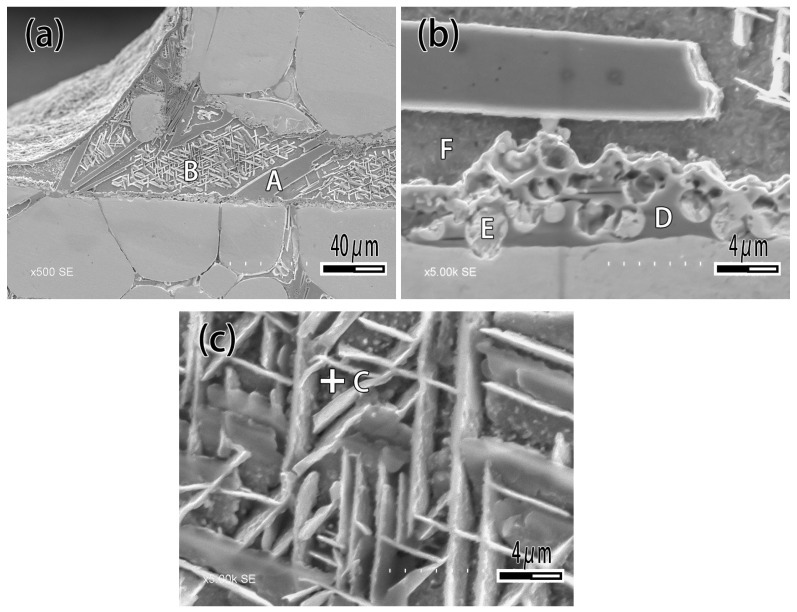
Microstructure of joint brazed by CuAg-10Ti filler metal. (**a**) Joint; (**b**) Interface; (**c**) Brazing central zone.

Figure 7 exhibits the microstructure of brazing joint with different Ti content. It can be seen that when the Ti content is 1wt.% (Figure 7a), the microstructure of brazing zone consisted of α-Ag and β-Cu, which is not significantly different from joint brazed by CuAg binary alloy. With the Ti content increasing (Figure 7b,c), the rectangular and acicular structure appeared, and a black reaction layer was produced at the interface. With the Ti content increasing (Figure 7c), the number of rectangular structures was raised. When the Ti content is 10 wt.% (Figure 7d), the filler metal reacted strongly with the binder phase of tungsten heavy alloy, and a phenomenon similar to intergranular infiltration occurred, so that the tungsten particles entered the filler metal as shown in Figure 7d. Figure 8 is the detail of microstructure of joint with 10% Ti content. As Figure 8 exhibits with EDS analysis (The results are shown in Table 3), the rectangular structure is the Ti_2_Cu phase marked A, and the acicular phase is the Ti_3_Cu_4_ phase marked B, as shown in Figure 8a. The Ag element mainly exists in the gap between acicular Ti_3_Cu_4_ phase (point C). The reaction layer at the interface is Ti_2_Cu phase too. Several tungsten particles are intercalated in the reaction layer such as point E, and occasionally there are solid solution of α-Ti around the tungsten particles (Figure 8b).

**Figure 9:**
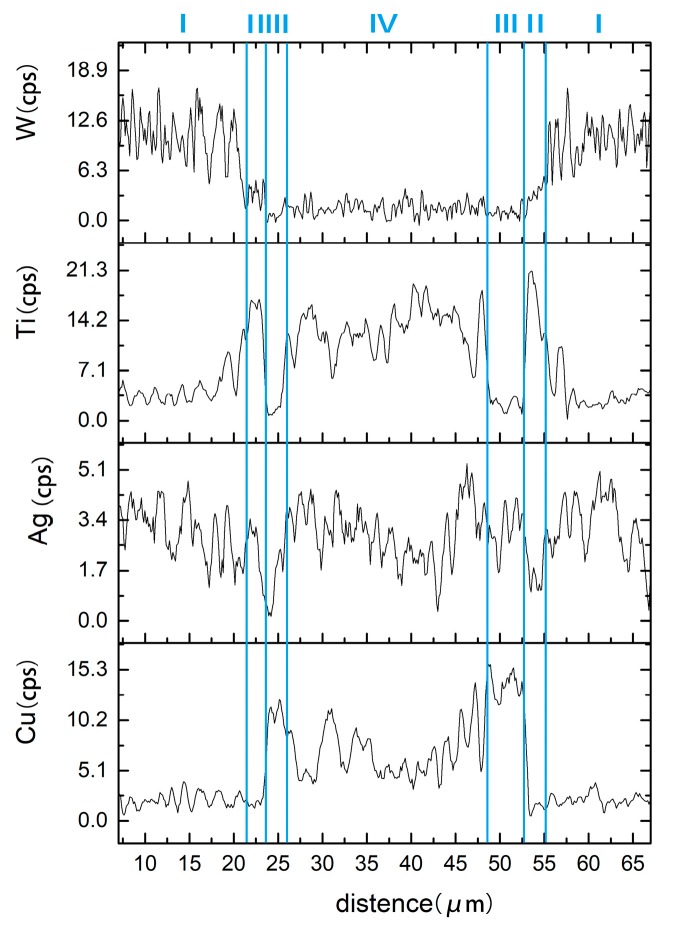
Line scan across the brazed joint.

**Figure 10:**
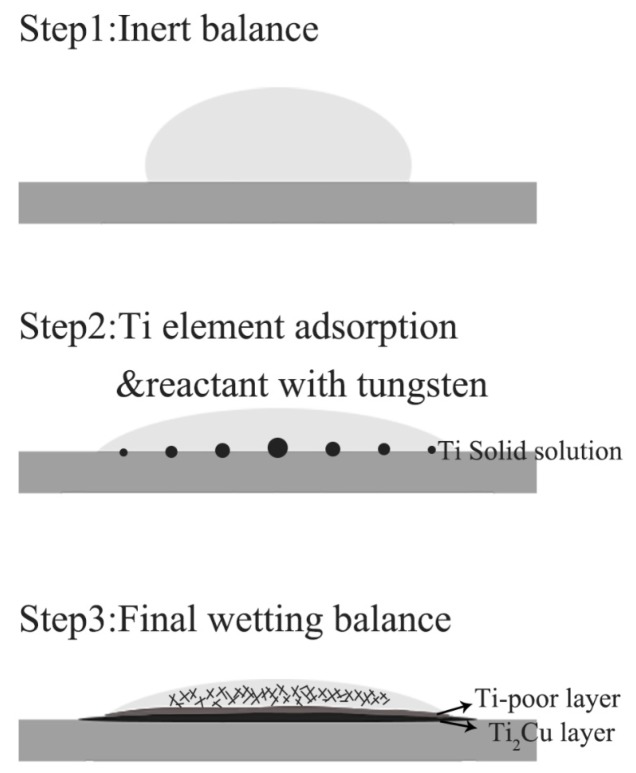
Wetting model.

**Figure 11:**
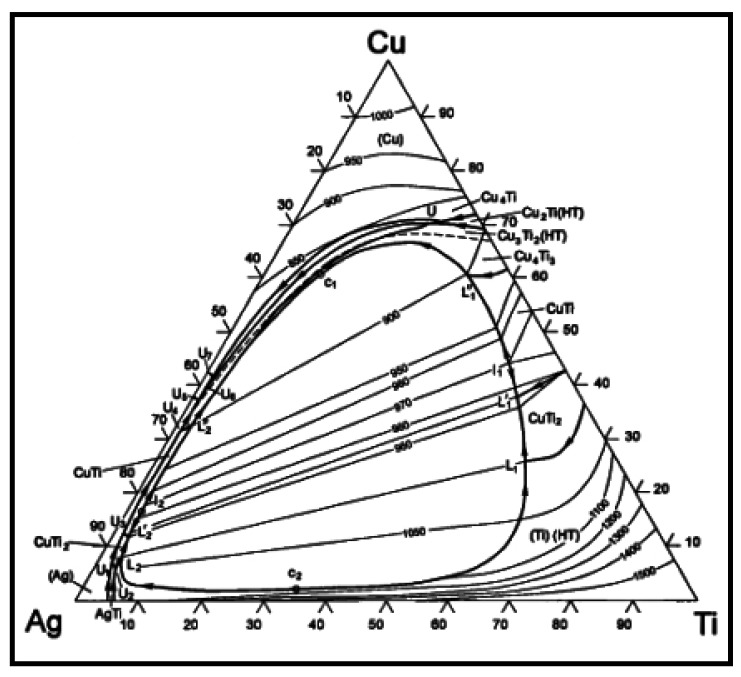
Ag-Cu-Ti ternary alloy phase diagram.

The element content of line scanning analysis of brazing joint is shown in Figure 9. The joint is divided into parts I, II, III and IV. Part I is base metal. Part II is a Ti-rich layer, and the W element content of line scanning forms an interlude step in this layer, W element dissolves more in this layer. Part III is Cu-rich layer, in which small amount of another element dissolved. Part IV is the center of brazing zone. This process of wetting and jointing can be regarded as an interface adsorption model of an active element, as shown as Figure 10. According to an Ag-Cu-Ti ternary alloy phase diagram (Figure 11), the filler metal should be separated into two liquid phases which are incompatible after melting into liquid, and one is rich in Cu and Ti, the other one is rich in Ag. Ti is active element which is able to react with W, so Ti is preferentially adsorbed on the surface of tungsten heavy alloy, while a Ti_2_Cu layer is formed at the interface, with a small amount of W diffusion inside. Concurrently, a Ti-poor area containing plenty of copper formed near the Ti_2_Cu layer. At the center of the brazing zone, Cu and Ti element remained form the acicular Ti_3_Cu_4_ phase, and the Ti_3_Cu_4_ phase separates out with a Ag-rich phase.

### 3.3. Mechanical Properties

**Figure 12:**
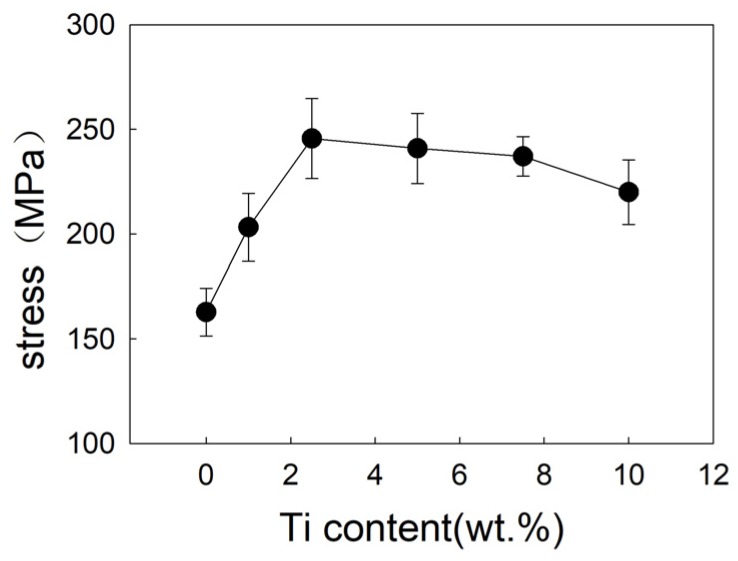
Influence of Ti content on shear strength of brazed joint.

**Figure 13:**
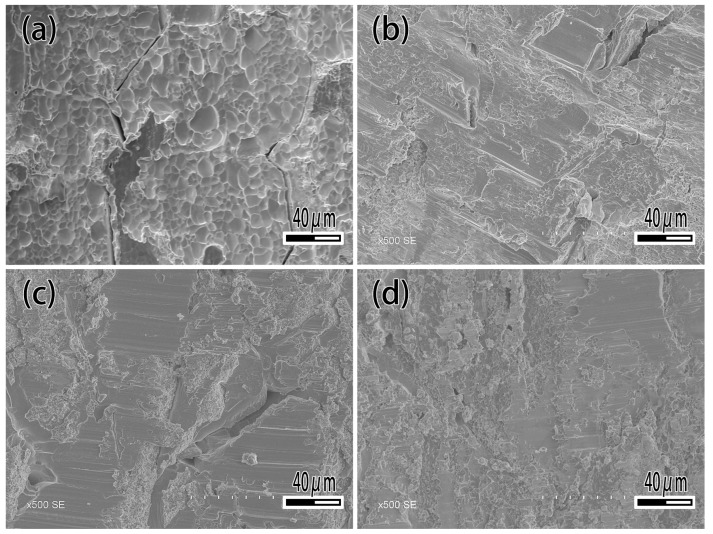
Fracture morphology of brazed joint. (**a**) Ti 1 wt.%; (**b**) Ti 2.5 wt.%; (**c**) Ti 7.5 wt.%; (**d**) Ti 10 wt.%.

Figure 12 and Figure 13 show the shear strength and fracture morphology of the joint with different Ti content brazed at 1050 °C for 10 min. In the test range, the shear strength of the joint is 162.8 MPa with no Ti. As the increasing of Ti content occurs, the shear strength of joint increases, which reached a maximum of 245.6 MPa while the Ti content is 2.5 wt.%. When continuing to add the Ti element, the shear strength of brazed joint decreased slightly, decreasing to 240.9 MPa at 5 wt.% Ti content, and decreasing to 220 MPa at 10 wt.% Ti content. As shown in Figure 13, the joint fractured mixed the characteristics of fragility and ductile. When Ti content is 1 wt.% (Figure 13a), it can be seen that the joint is crystalline fracture, which means there is joint insecurity. The diffusion of Ti and the formation of the reaction layer improve the shear strength of joint and reaches the highest level at 2.5 wt.%. Then an excessive Ti element causes the dissolution of the base metal into the filler metal, resulting in the inclusion of tungsten particles in the reaction layer and reducing shear strength of the joint.

**Figure 14:**
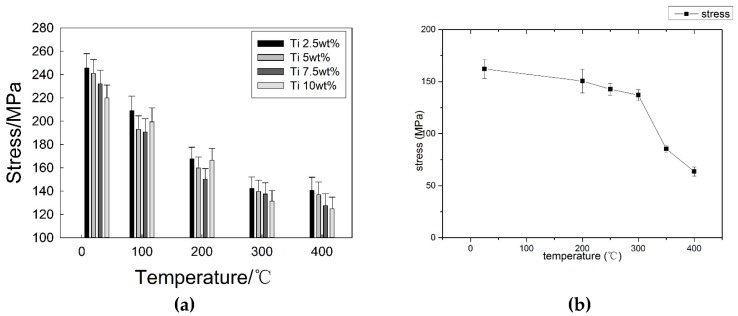
Influence of testing temperature on Shear Strength of brazed Joint. (**a**) CuAgTi filler metal; (**b**) without Ti.

As a die-casting mold, tungsten heavy alloy needs to withstand the repeated scouring of high temperature liquid, and high temperature properties are necessary for tungsten heavy alloy. Therefore, it is important to study the influence of temperature on the shear strength of a brazed joint. Figure 14 is the result of a high temperature performance test. Figure 14a shows the influence of temperature on the shear strength of a joint with different Ti contents, and Figure 14b is a comparison of joints brazed by CuAg filler metal without Ti. The shear strength of joint brazed by CuAg filler metal at room temperature is 162.2 MPa, however it reduces to 63.6 MPa at 400 °C, decreased by 60.8%. It can be seen that the Ti element can promote the shear strength of joint at room temperature and high temperature. After adding Ti element, the shear strength of joint at 400 °C has a range from 124.8 to 142.8 MPa. However, there is little difference in high temperature performance of the brazed joint with a different Ti content.

## 4. Conclusions

In order to solve the two problems of controlling brazing temperature and intermetallic compound joint strength reduction caused by intermetallic compounds in the brazing of tungsten-based powder alloy, we chose Cu-30Ag as a base filler metal and added the Ti element to promote its interaction with base metal, then studied the influence of Ti on solder microstructure, joint structure and mechanical properties, concluding as follows.

The Ti element influences the microstructure of the filler metal. When the Ti content is less than 2.5 wt.%, the microstructure of the CuAgTi filler metal consists of α-Cu, β-Ag and Ti solidus solution which were concentrated on the boundary between α-Cu and β-Ag. With the addition of the Ti element, the rectangular Ti_3_Cu_4_ phase appeared in the filler metal. Ti element can promote the interface reaction between filler metal and base metal. During the brazing process, Ti elements are concentrated at the interface near base metal, forming a Ti-rich Ti_2_Cu layer. Then a Cu-rich/Ti-poor layer formed near the Ti_2_Cu layer. Finally, the acicular Ti_3_Cu_4_ phase separated out together with the silver-rich phase.

Due to the promoting of the Ti element on the interfacial reaction, Ti has a great effect on improving the wettability of CuAgTi filler metal; with the increase of the Ti element, the spreading area increases from 120.31 mm^2^ to 283.87 mm^2^, while the area is increased by 135.9%. Excessive Ti element promotes the dissolution of the base metal and reduces the strength of the joint. When the Ti content is 2.5 wt.%, the shear strength of brazed joint at room temperature reaches the highest level, 245.6MPa. Meanwhile, the shear strength at 400 °C is 142.2 MPa, which is 100% higher than that without Ti. A significant difference in high temperature performance of the brazed joint with different Ti content has not been observed.

According to the test results, the wettability, mechanical properties and high temperature strength of the brazing filler metal are the best levels at Ti 2.5 wt.%, while Cu-30Ag-2.5Ti is the most suitable component. Based on this, we hope to further study the effects of other active elements such as Zr or Nb in the next work and study their interaction with Ti, optimize the composition of the brazing alloy, and obtain a brazed joint with better mechanical properties.

## Figures and Tables

**Table 1:** Chemical compositions of filler metal (wt.%).

Element	1	2	3	4	5	6
**Cu**	70	69	67.5	65	62.5	60
**Ag**	30	30	30	30	30	30
**Ti**	-	1	2.5	5	7.5	10

**Table 2:** EDS analysis of CuAgTi filler metal (at.%).

Point	Cu (at.%)	Ag (at.%)	Ti (at.%)	Phase
**A**	12.21	5.74	82.05	α-Ti
**B**	54.47	3.69	41.84	Ti_3_Cu_4_
**C**	53.12	1.96	44.92	Ti_3_Cu_4_
**D**	59.04	1.03	39.93	Ti_3_Cu_4_
**E**	92.85	5.73	1.42	α-Cu
**F**	15.10	84.49	4.41	β-Ag

**Table 3:** EDS analysis of brazed joint.

Point	Cu (at.%)	Ag (at.%)	Ti (at.%)	W (at.%)	Phase
**A**	53.47	3.99	40.67	1.87	Ti_3_Cu_4_
**B**	62.44	2.04	30.94	4.57	Ti_2_Cu
**C**	8.73	90.17	1.10	-	β-Ag
**D**	27.35	1.34	63.91	7.39	Ti_2_Cu&α-Ti
**E**	0.67	0.22	3.34	95.77	W granule
**F**	71.56	6.99	3.24	2.47	α-Cu
